# “Mitochondrial Toolbox” – A Review of Online Resources to Explore Mitochondrial Genomics

**DOI:** 10.3389/fgene.2020.00439

**Published:** 2020-05-08

**Authors:** Ruaidhri Cappa, Cassio de Campos, Alexander P. Maxwell, Amy J. McKnight

**Affiliations:** ^1^Centre for Public Health, Institute of Clinical Sciences B, Queen’s University Belfast, Royal Victoria Hospital, Belfast, United Kingdom; ^2^School of Electronics, Electrical Engineering and Computer Science, Queen’s University Belfast, Belfast, United Kingdom

**Keywords:** association, genome, methylation, mitochondria, resource, SNP, toolbox

## Abstract

Mitochondria play a significant role in many biological systems. There is emerging evidence that differences in the mitochondrial genome may contribute to multiple common diseases, leading to an increasing number of studies exploring mitochondrial genomics. There is often a large amount of complex data generated (for example *via* next generation sequencing), which requires optimised bioinformatics tools to efficiently and effectively generate robust outcomes from these large datasets. Twenty-four online resources dedicated to mitochondrial genomics were reviewed. This ‘mitochondrial toolbox’ summary resource will enable researchers to rapidly identify the resource(s) most suitable for their needs. These resources fulfil a variety of functions, with some being highly specialised. No single tool will provide all users with the resources they require; therefore, the most suitable tool will vary between users depending on the nature of the work they aim to carry out. Genetics resources are well established for phylogeny and DNA sequence changes, but further epigenetic and gene expression resources need to be developed for mitochondrial genomics.

## Introduction

**FIGURE 1:**
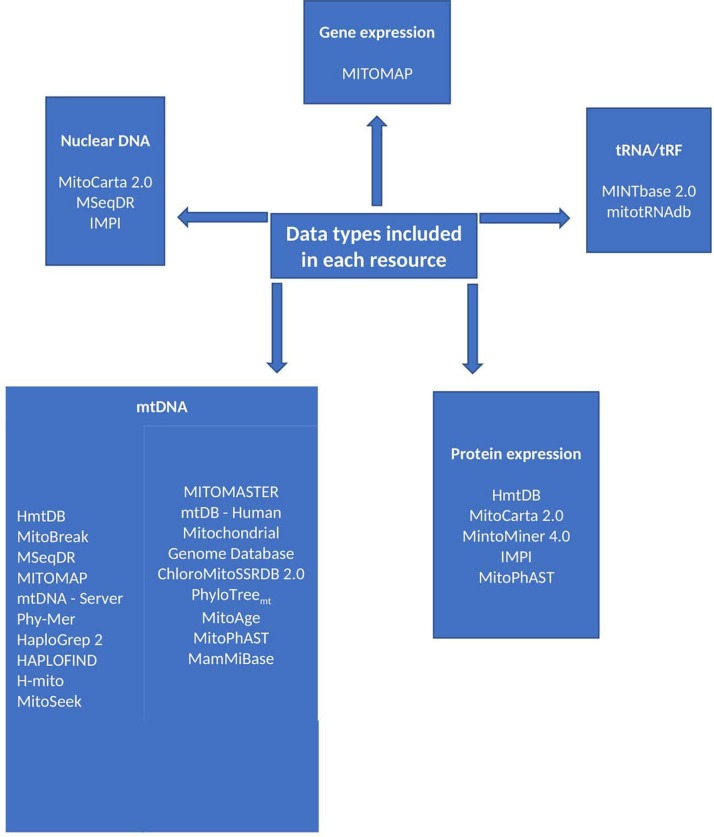
Resource data types. The discussed resources include a variety of data types as summarised in this figure. The type of data included in a resource is liable to influence the choice of resource.

**FIGURE 2:**
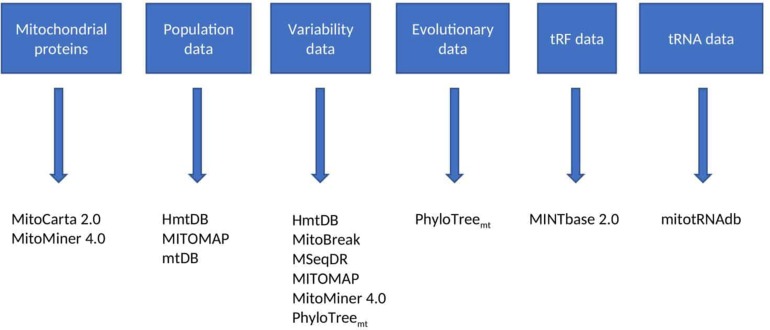
Reference databases. This figure lists the discussed resources which contain reference databases, and the type of data contained within these resources (some contain more than one type of data). The type of data desired by the user will determine which resources are most appropriate for the study in question.

In this review, we discuss the role of the mitochondria in relation to disease, with a focus on mitochondrial genomics, and review the bioinformatic tools currently available for the analysis of the mitochondrial genome. This brief overview highlights the importance of the mitochondrial genome from a scientific and clinical perspective, and also provides readers with signposts to the bioinformatics tools currently available. We discuss and compare the capabilities offered by a variety of bioinformatics software (Figures 1, 2), helping readers to make an informed choice as to which tools would best suit their needs. We also discuss challenges relating to the field of mitochondrial genomics and discuss bioinformatics capabilities which are currently lacking. This may provide perspective for researchers seeking to develop new bioinformatics tools by informing them of the current gaps in the market, and therefore may provide inspiration for the development of new tools which would enable researchers to investigate the mitochondrial genome further. This review extends a mini-review previous published by Bris et al. (2018).

Mitochondrial diseases affect more than 10 per 100,000 individuals (including mitochondrial disorders caused by mutations in either the mitochondrial or nuclear genomes) (Gorman et al., 2015). Developing and implementing effective treatments and preventative strategies for mitochondrial disease may improve the health of a substantial portion of the population (Gorman et al., 2015).

Identifying pathogenic mutations in mitochondrial DNA (mtDNA) may also provide more effective diagnostic tools in addition to aiding disease prevalence studies, which can be used to inform healthcare budgets and public health strategies (Gorman et al., 2015). Correctly diagnosing severe pathogenic mutations in mtDNA is also necessary when considering techniques such as *in vitro* fertilisation to prevent such mutations being passed on to future generations (Gorman et al., 2015). This has been demonstrated in proof of concept studies, which have used an approach of transplanting pronuclei soon before the first mitotic division occurred; however, it has been found that normally fertilised zygotes are unable to satisfactorily tolerate this approach (Hyslop et al., 2016). An alternative method has been developed in which pronuclei are transplanted just after meiosis has been completed prior to the first mitotic division. This method did appear to be successful as development continued successfully to blastocyst stage (Hyslop et al., 2016). Using this method, mtDNA carryover was reduced to less than 2% in 79% of pronuclear transplantation (PNT) blastocysts (Hyslop et al., 2016). This avoided the progressive increase in heteroplasmy which has been observed when mtDNA carryover levels are at 4% or higher (Hyslop et al., 2016). The PNT method could reduce the likelihood of mitochondrial disease occurring, however, there is no guarantee that disease would not occur (Hyslop et al., 2016).

Another method of avoiding transmission of mtDNA disease to offspring is the replacement of oocyte maternal mtDNA. The mother’s oocytes mutant mtDNA can be replaced using a spindle transfer method, resulting in the development of embryos which contained over 99% donor mtDNA (which lacked harmful mutations) (Kang et al., 2016). Embryonic stem cells derived from such embryos stably maintain the donor mtDNA in the majority of cases, however, in some cases the donor mtDNA is gradually lost and the cells reverted to the (disease causing) maternal haplotype (Kang et al., 2016). It is possible that a matching paradigm could be used in order to select compatible donor mtDNA for use in mitochondrial replacement therapies or techniques (Kang et al., 2016).

Such as a paradigm could be informed by the finding that donor mtDNA compatibility is related to replication efficiency, and also the identification of a polymorphism which may be related to preferential replication of certain mtDNA haplotypes (Kang et al., 2016).

Widely used methods of genetic engineering, such as the CRISPR-Cas9 system, are currently not widely employed to modify the mitochondrial genome, although the genome editing tool CRISPR/Cas9 can localise specifically to the mitochondria (using a mitoCas9), and the system can cleave mtDNA at specific loci (Jo et al., 2015). However, there is a lack of other studies which demonstrate successful and specific modification of mtDNA using CRISPR/Cas9. Furthermore, employing CRISPR/Cas9 to modify mtDNA would require the systems guide RNA to be imported into the mitochondria. This is somewhat controversial because there is no accepted mechanism by which RNA may be imported into mammalian mitochondria, and it is not accepted as to what function such molecules would serve if imported. While certain studies suggested that RNAs imported into the mitochondria may serve functions such as mitochondrial RNA processing (Chang and Clayton, 1987; Rosenblad et al., 2006), this was contradicted by other studies (Kiss and Filipowicz, 1992; Jacobson et al., 1995; Wanrooij et al., 2010). It has been suggested that mammalian mitochondria could function normally without the need for endogenous RNA import (Gammage et al., 2018) although it has been shown that a range of RNAs can be artificially targeted to the mitochondria (Wang et al., 2012).

A major limitation of current genetic engineering techniques in relation to modifying the mitochondrial genome is the inability of these tools to introduce the desired modifications in a homoplasmic manner (Verechshagina et al., 2019). Current tools shift the mtDNA heteroplasmy level toward a more desirable state (Verechshagina et al., 2019). Changes in mitochondrial heteroplasmy may have transcriptomic, epigenomic, and metabolomic consequences, such as altered histone modifications and changes to the redox state (Kopinski et al., 2019). Therefore, it is possible that the use of existing genetic engineering techniques to modify the mitochondrial genome in a heteroplasmic manner may have unintended and possibly negative consequences. In order for a tool to be considered a reliable means of modifying the mitochondria genome it would be required to induce the desired alterations in a specific and homoplasmic manner (Verechshagina et al., 2019).

Therefore it is generally accepted that there are no reliable methods for modifying the human mitochondrial genome at present (Klucnika and Ma, 2020).

Alternative approaches have achieved some success, such as approaches which utilise mitochondrially targeted zinc finger-nucleases. Such systems are engineered to specifically bind to DNA sequences of interest (Urnov et al., 2005). After binding a double strand break is induced which stimulates homologous recombination between the target DNA and the DNA donor (Urnov et al., 2005). Mitochondrially targeted zinc finger-nucleases have been demonstrated and may be effective due to mtDNA typically being heteroplasmic, i.e., one cell will contain numerous mitochondria, which are likely to represent a mixture of mutant and wild type mtDNA (Gammage et al., 2014). Disease is typically only observable if the ratio of mutant mtDNA crosses a certain threshold [which can be between 70 and 90% (Mattiazzi, 2004)], therefore systems which selectively eliminate mtDNA possessing pathogenic mutations allows the cell to achieve a desirable heteroplasmic ratio which does not result in a pathogenic state (Gammage et al., 2014).

An additional method of mtDNA modification involves mitochondrially targeted transcription activator-like effector nucleases (mitoTALENs), which have been demonstrated to cleave specific mtDNA sequences, therefore providing a means of eliminating mtDNA possessing pathogenic mutations, and bringing the target cells to a more desirable heteroplasmic state (Hashimoto et al., 2015). Also, mitochondria targeted restriction endonucleases have been demonstrated in a proof of concept study in which mouse mtDNA was reduced in the germline in order to prevent BALB or NZB haplotype variants being transmitted to offspring (Reddy et al., 2015).

It has been suggested that any method which involves cleaving mtDNA, such as those discussed, may cause the cellular mtDNA content to drop below functional levels, which would prevent the clinical application of such methods (Moraes, 2014). However, eliminated mtDNA is replaced by an uncharacterised mechanism by which mtDNA copy number is maintained (Carling et al., 2011), and research has suggested that heart, liver (Bacman et al., 2010), and skeletal muscle (Bacman et al., 2012) can tolerate the temporary drops in mtDNA levels before the cells are repopulated with wild type mtDNA. This has not been demonstrated that all cell types, and the evidence is not conclusive (Patananan et al., 2016). Despite the progress that has been made, more research will be required before it is possible to employ mainstream methods of genetic engineering to modify the mitochondrial genome. Mitochondrial genomic alterations are increasingly recognised as contributing to common, complex diseases such as cardiovascular disease (CVD) (Meyers et al., 2013; Bray and Ballinger, 2017) and kidney disease (Swan et al., 2015; Connor et al., 2017). These genomic alterations may be at the level of MtDNA sequence (Bray and Ballinger, 2017), or epigenetic modifications (Baccarelli and Byun, 2015), that result in gene expression changes (Devall et al., 2014) influencing the presentation and progression of human diseases.

## Mitochondrial Genome and Common Diseases

Mitochondria possess their own genome which is used for the production of some of the proteins they require to function (Meiklejohn et al., 2013). Complementing this, many of the proteins required by mitochondria are encoded by the nuclear genome (Meiklejohn et al., 2013). In humans, mitochondrial genomes (MtDNA) are circular and are 16,569 base pairs long, and include 37 genes, which encode 13 proteins that are required for oxidative phosphorylation and the electron transport chain. The human mitochondrial genome also encodes RNAs which are involved in the translation of the proteins encoded by the mitochondrial genome (Taanman, 1999).

Mutations in the mitochondrial genome can negatively affect mitochondrial function, which may influence the onset or progression of diseases such as ankylosing spondylitis, multiple sclerosis, ischaemic stroke, etc. (Hudson et al., 2014). A single mutation in MtDNA may be related to the development of multiple disorders, since a single mutation can affect the normal functioning of the cell/body in numerous ways (Hudson et al., 2014). For example, the m.310 variant (of the mitochondrial genome) is associated with ankylosing spondylitis, multiple sclerosis, ischaemic stroke, Parkinson’s disease (PD), and psoriasis, and the m.497 variant is associated with multiple sclerosis, ischaemic stroke, and primary biliary cirrhosis (Hudson et al., 2014).

Oxidative phosphorylation performed by mitochondria results in the production of reactive oxygen species (ROS). These ROS have vital physiological roles in a range of intracellular signalling pathways (Finkel, 2011) including those related to the inflammatory response (Bulua et al., 2011) and insulin sensitivity (Leloup et al., 2009). However, ROS may also cause DNA damage and potentially induce somatic mutations (Douglas et al., 2014). It has been suggested that the mitochondria may be more susceptible to somatic mutations than nuclear DNA since the mitochondrial genome likely has greater contact with ROS (Douglas et al., 2014). Mitochondrial damage may also cause the respiratory chain to become impaired (Kalyanaraman, 2013) and may result in increased electron leakage from the respiratory chain (Granata et al., 2015), which may increase the likelihood of ROS forming (Kalyanaraman, 2013; Granata et al., 2015). ROS induce DNA mutations by causing redox reactions although particular mechanism of biological damage occurring depends on the particular type of ROS involved (Imlay and Linn, 1988). Often the ROS are intermediate compounds produced during the reduction of oxygen during aerobic metabolism (Marnett, 2000). These oxygen radicals either attack the DNA bases or the deoxyribose backbone directly, or alternatively react with biological molecules like lipids, which in turn generate reactive molecules which then interact with DNA bases (Marnett, 2000).

The damage caused by ROS generated from dysfunctional mitochondria is a contributing factor to a number of diseases. For example, the mtDNA T8993G (NARP) mutation has been shown to inhibit oxidative phosphorylation in both NARP (neuropathy, ataxia, and retinitis pigmentosa) (Holt et al., 1990) and MILS (maternally inherited Leigh’s syndrome) (Santorelli et al., 1993) resulting in increased ROS levels and free radical damage. It has been observed that mitochondrial respiration and adenosine triphosphate (ATP) synthesis can be improved in cells possessing the mtDNA T8993G (NARP) mutation by treating the cells with antioxidants, suggesting that the mechanism by which the mutation causes damage is at least in part due to ROS (Mattiazzi, 2004). ROS generated by mitochondria have been shown to induce post-translational modifications of mitochondrial proteins, which results in mitochondrial dysfunction in lipotoxic cardiomyopathy (Tsushima et al., 2018). Increased ROS levels are correlated with mitochondrial network fragmentation and may also be associated with decreased mitochondria size (Tsushima et al., 2018). The observed correlations are reinforced by the finding that the effects of increased ROS levels can be alleviated by SOD2 overexpression (Tsushima et al., 2018), which is known to mediate superoxide detoxification (Murphy, 2009).

The Warburg effect refers to the fact that cancer cells up-regulate glycolysis and predominantly perform lactate fermentation rather than mitochondrial oxidative phosphorylation as is the case in non-cancerous cells (Lu et al., 2015). This allows cancer cells to avoid excess ROS levels that would otherwise be generated by mitochondrial oxidative phosphorylation, which results in cancer cells gaining increased resistance to anoikis and therefore increased ability for metastasis, increasing the ability of the cancer to spread (Lu et al., 2015).

As well as being directly related to disease, mutations in mtDNA can interact with the nuclear genome, which may affect the risk of developing disease (or multiple diseases) (Hudson et al., 2014; Swan et al., 2015).

Most mtDNA mutations are heteroplasmic, i.e., these mutations are not found in every mitochondrion in every cell of the affected individual, many of the mitochondria in these cells may have normal genomes. These heteroplasmic mutations may have pathogenic potential in healthy individuals (Ye et al., 2014).

## Mitochondrial Genome in Population Genetic Studies

mtDNA is usually excluded from population genetic studies because it is maternally inherited, is subject to a higher mutational rate than nuclear DNA, and because recombination of mtDNA does not take place; however, mtDNA can be used to investigate genetic relationships between individuals within populations over time and has been used as an indicator of accelerated biological ageing (Kivisild, 2015).

The mitochondrial genome can be sequenced using next generation sequencing (NGS) to detect mutations, the heteroplasmy of point mutations can be quantified, and deletion breakpoints can be determined. NGS is highly effective for diagnosing mtDNA related diseases (Palculict et al., 2016).

The data generated *via* NGS is large and complex, and therefore analysis of such data requires specialist software/computational tools and databases (Belmont and Shaw, 2016).

## Mitochondrial Genome and Metabolic Pathways

Mitochondria have functions other than the production of ATP including production of macromolecules and in activating signalling pathways. These mitochondrial functions may be related to the development of cancer, therefore it is possible that cancer treatments could be developed which would affect mitochondrial metabolism (Weinberg and Chandel, 2015). For example, the mitochondrial folate pathway is significantly upregulated in cancer tissues; in particular, the MTHFD2 enzyme is highly expressed in a number of cancer types, and is correlated with lower survival rates among breast cancer patients (Nilsson et al., 2014).

Mitochondrial genomic mutations can occur spontaneously, and can accumulate as an individual grows older (Michikawa et al., 1999). These mutations can result in defective mitochondrial β-oxidation, which causes an increase in the levels of fatty acids (Petersen et al., 2003). This is a contributing factor to fatty acid induced insulin resistance, and is an example of how mitochondrial genomic mutations related to a metabolic pathway can be involved in the pathogenesis of disease (Petersen et al., 2003). Mutations in the mitochondrial genome may also reduce the ability of mitochondria to generate ATP; the lack of ATP would inhibit the ability to transport insulin and glucose, which could play a role in the development of diabetes (Kelley et al., 2002). Mitochondria in the muscles of patients suffering from type 2 diabetes can be smaller in both size and number, and displayed reduced activity, than those found in healthy controls, suggesting that mitochondrial defects play a role in type 2 diabetes (Kelley et al., 2002).

Haem, a cofactor consisting of Fe^2+^ contained within the organic compound porphyrin that is required for the synthesis of haemoglobin, is synthesised within mitochondria (Atamna, 2004). Inhibition of haem metabolism is related to the onset of oxidative stress, mitochondrial decay, and increased iron levels, which are known to be associated with ageing (Atamna, 2004). Dysfunctional haem metabolism has the potential to play a role in diseases related to ageing, and also may play a role in iron deficiency (Atamna, 2004). Since haem synthesis occurs in mitochondria, this is an example of how a mitochondrial metabolic pathway other than ATP production can potentially play a role in disease (Atamna, 2004).

Two mutations within the mtDNA gene COX I (complex IV of the electron transport chain) at positions 6742 (T>C) and 6721 (T>C) were identified in individuals suffering from acquired idiopathic sideroblastic anaemia (AISA), suggesting these are pathogenic mutations which cause respiratory chain dysfunction by inhibiting the reduction of Fe^3+^ to Fe^2+^ (Gattermann et al., 1997). Mutations in subunit I of cytochrome oxidase may also be relevant, given the involvement of haem with cytochrome oxidase, as mutations at position 6742 and 6721 of this gene result in amino acid changes, which are otherwise highly conserved sites (Broker et al., 1998).

Mitochondria require cholesterol in order to perform functions such as the synthesis of bile acids. The build-up of cholesterol in the mitochondria may be related to disease, for example increased cholesterol levels results in oxidative stress and cell death, which promotes the transition from simple steatosis to steatohepatitis, which may progress to hepatocellular carcinoma (García-Ruiz et al., 2016). Mitochondrial cholesterol also plays a role in resistance to apoptosis and insensitivity to chemotherapy in hepatocellular carcinoma (García-Ruiz et al., 2016). It may be possible to modulate the progression from steatohepatitis to hepatocellular carcinoma by taking steps to regulate mitochondrial cholesterol trafficking (García-Ruiz et al., 2016).

## Mitochondria in Cancer Metabolism

As discussed, cancer cells predominantly up-regulate glycolysis and mainly perform lactate fermentation as opposed to oxidative phosphorylation (Lu et al., 2015), but they do require mitochondrial activities for survival (Cheng et al., 2011). The absence of mitochondrial oxidative phosphorylation in cancer cells may provide them with survival advantages; this results in an increase in NADH levels, which results the inactivation of PTEN through a redox modification mechanism and activation of the Atk survival pathway, which provides cancer cells with drug resistance and improved ability to survive during hypoxia (Pelicano et al., 2006).

Evidence for the involvement of the mitochondrial genome in the pathogenesis of cancer includes the association of certain inherited mtDNA variations with increased susceptibility to cancer, and a number of cancers are associated with mtDNA haplogroups, for example individuals with the M7b2 haplogroup display an increased risk of developing hematopoietic cancer and leukaemia (Minocherhomji et al., 2012). The U haplogroup is associated with prostate and renal cancers (Booker et al., 2006); the A10398G and T16519C polymorphisms of mtDNA are associated with breast cancer (Bai et al., 2007).

Certain mitochondrial metabolites are associated with cancer onset such as 2-hydroxyglutarate in the case of gliomas (Dang et al., 2009). Mutations in *FH* cause fumarate hydratase deficiency which may play a role in hereditary leiomyomatosis and renal cell cancer (Wei et al., 2006; Ternette et al., 2013). The succinate dehydrogenase subunit genes also display mutations in cancer patients, for example *SDHA* displays mutations such as p.Gln54X, p.Thr267Met, and c.1663+3G>C, which are present in Succinate Dehydrogenase–deficient Gastrointestinal Stromal Tumours (Dwight et al., 2013). *SDHB* mutations have been identified in Familial renal cell carcinoma patients (Ricketts et al., 2008), *SDHC* mutations in cases of autosomal dominant paraganglioma type 3 (Niemann and Muller, 2000) and a case of epithelial thyroid cancer combined with carotid paraganglioma (Zbuk et al., 2007). *SDHD* displays reduced expression and various mutations in individuals affected by colorectal and gastric cancers (Habano et al., 2003). Mitochondrial ROS are associated with cancerous DNA mutations (Sabharwal and Schumacker, 2014), such as mutations in mitochondrial *IDH2* in gliomas (Yan et al., 2009) and myeloid leukaemia (Marcucci et al., 2010). Cancer cells also display various mutations in nuclear encoded mitochondrial genes such as *IDH1*, i.e., the point mutation Arg132-to-His (R132H) (Balss et al., 2008; Bleeker et al., 2009; Ichimura et al., 2009; Sanson et al., 2009). All cancerous *IDH1* and *IDH2* mutations may display the common feature of causing the conversion of a-ketoglutarate to 2-hydroxyglutarate *via* neomorphic enzyme activity (Ward et al., 2010).

Mitochondria may be involved in the activation of cancerous signalling pathways, such as mitogen activated protein kinase, which may regulate the G1 to S phase transition of the cell cycle (Meloche and Pouysségur, 2007). Also, mutations in may result in KRas included mitochondrial oxidative stress which results in the upregulation of epidermal growth factor signalling, which results in the formation of precancerous lesions in the pancreas (Liou et al., 2016).

Regulated cell death (apoptosis) is dependent upon the mitochondria (Zamzami et al., 1995, 1996); cancer cells typically do not undergo apoptosis in the normal fashion due to changes in the mitochondria (Hanahan and Weinberg, 2011). Mitochondrial outer membrane permeabilisation (MPTP), which may be caused by the mitochondrial permeability transition pore (MPTpore) (although the molecular make-up of the MPTP is not understood) (Mathupala et al., 2006) appears to play a significant role in apoptosis and in cancerous cells appear resistant to apoptosis (Certo et al., 2006; Czabotar et al., 2014). Cancerous cells that are resistant to apoptosis often display overexpressed Bcl-2 (a protein which regulates cell death) (Certo et al., 2006; Czabotar et al., 2014). Although Bcl-2 would typically bind with ABT-737, resulting in the release of sequestered pro-apoptotic proteins and therefore cell death, many cancer cells appear insensitive to ABT-737, possibly because ABT-737 cannot target the protein Mcl-1 (van Delft et al., 2006). Mitochondrial permeability transition (MPT) is also involved in causing apoptosis. Cancer cells have been found to display inhibition of MPT, due to the activation of mitochondrial ERK via a signalling axis that includes cyclophilin D and GSK-3, which results in the desensitisation of cancer cells to the MPTpore (Rasola et al., 2010).

## Epigenetics and Gene Expression in Relation to the Mitochondrial Genome and Disease

Epigenetic processes can be a mechanism by which environmental signals contribute to biological changes, which may result in disease (Devall et al., 2014). Epigenetic changes occur within the mitochondrial genome, and may play a role in diseases associated with mitochondrial dysfunction, such as Alzheimer’s disease (AD) (Devall et al., 2014). Mitochondrial dysfunction occurs in AD in the brain and white blood cells, but the molecular mechanisms by which this occurs are not fully understood (Devall et al., 2014). A number of regions of the genome are consistently differentially methylated, some of which are specific to certain tissues in the brain which are associated with AD pathology (Devall et al., 2014).

Mutations in the mitochondrial genome may result in epigenetic changes which may cause instability in the nuclear genome (Minocherhomji et al., 2012). Mutations of mtDNA, such as changes in mtDNA copy number, are associated with tumour development (Minocherhomji et al., 2012). Mutations in mtDNA, can influence expression of nuclear genes involved in cell signalling, metabolism, and growth (Minocherhomji et al., 2012).

Gene expression from the mitochondrial genome is affected by both sequence dependent and sequence independent mechanisms. Variability in mtDNA sequence or content has a significant effect on the variability of gene expression, possibly accounting for around 50% of the variability in protein levels (Guantes et al., 2015). This variability contributes to mitochondrial impacts on transcription and translation apparatus content and activity, which influences mRNA quantity, translation, and alternative splicing, which consequently affects the cellular phenotype (Guantes et al., 2015). Sequence independent mechanisms of gene expression include the stochastic nature of the gene expression process, i.e., it behaves randomly according to a certain probability distribution, resulting in variation of gene expression between genetically identical cells in identical environments (Munsky et al., 2012). Variation in the observed phenotype may occur due to the random aspects of gene expression without altering the nucleotide sequence; this is a significant aspect of developmental processes, cell differentiation, and cancer (Guantes et al., 2015). The amount of energy made available for gene expression can be influenced by factors such as the tissues demand for energy, the number of mitochondria present, and cell stress (Muir et al., 2016). If mitochondrial function is inhibited, due to genomic variation or other factors, then there will be a significant effect on nuclear gene expression due to a shortage of energy, which results in increased risks of conditions such as degenerative diseases, cancer, and age related illness (Guantes et al., 2015; Muir et al., 2016).

## miRNAs and Mitochondria

miRNAs are non-coding RNAs which act as negative regulators of gene expression, either by inhibiting translation or degrading target mRNA (Bartel, 2004). miRNAs may form in the cytoplasm before being transported into the mitochondria via carrier proteins such as Ago2 (Zhang et al., 2014), and may also be encoded by the mitochondrial genome; however, these miRNAs are not well understood (Barrey et al., 2011).

miRNAs can affect mitochondrial function by inhibiting the expression of proteins involved in mitochondrial transport. For example; the miRNA miR-141 has been shown to inhibit the expression of Slc25a3, a mitochondrial phosphate carrier (Baseler et al., 2012). Due to Slc25a3 inhibition there is a lack of phosphate available for use in ATP synthesis in the mitochondria. Therefore miR-141 can reduce the quantity of ATP synthesised (Baseler et al., 2012). This could potentially be a factor in the pathogenesis of type 1 diabetes, as it has been observed that miR-141 is more highly expressed in patients suffering from type 1 diabetes compared to non-diabetic individuals, and that this influences the levels of ATP in the heart of the diabetic patients (Baseler et al., 2012).

Another miRNA, miR-184, has been shown to inhibit the expression of Slc25a22, which is a mitochondrial glutamate carrier (Morita et al., 2013). Glutamate acts to induce insulin excretion, therefore when Slc25a22 expression is inhibited the level of insulin secreted by pancreatic β cells is reduced (Morita et al., 2013). Therefore miR-184 has the potential for involvement in the pathogenesis of diabetes by reducing the level of glucose-induced insulin secretion (Morita et al., 2013).

miR-181c is a nuclear encoded miRNA, which is found in mitochondria of heart cells (Das et al., 2017). miR-181c regulates the expression of the mitochondrial gene mt-*COX1*, which is involved in complex IV of the respiratory chain (Das et al., 2017). Overexpression of miR-181c has been shown to result in decreased expression of mt-*COX1*, and therefore reduced levels of the mt-COX1 protein, decreased complex IV remodeling, and an increase in the levels of ROS, which is potentially harmful as ROS are known to cause oxidative damage to cellular macromolecules (Das et al., 2017).

## Some tRFs Act Like miRNAs

All human tRNAs appear to generate numerous fragments, known as tRNA-derived RNAs (tRFs). The length and abundance of the fragments appears to be influenced by factors such as race and sex, as well as the genomic locus, tissue type, and diseases (Telonis et al., 2015b). Many small RNAs can be derived from tRNA. Mitochondrial tRNAs produce a disproportionately larger amount of tRFs than nuclear tRNAs (Telonis et al., 2019), and have a different length distribution compared to nuclear encoded tRNAs (Telonis et al., 2015b). tRNA fragments originating from the same anticodon do not display correlated abundances (Telonis et al., 2015b).

tRFs may be associated with Argonaute proteins, but not all tRFs bind with Argonaute (Venkatesh et al., 2015; Kuscu et al., 2018). The functions of tRFs include post-transcriptional gene repression (Kuscu et al., 2018). The tRNA-derived fragment CU1276 displays functional properties of a miRNA such as association with Argonaute proteins and the ability to repress mRNA transcripts in a sequence-specific manner (Maute et al., 2013). CU1276 appears to be relevant from the perspective of lymphoma pathogenesis given that CU1276 is not expressed in lymphoma cells despite being expressed in normal germinal centre B cells (Maute et al., 2013). CU1276 has been demonstrated to repress *RPA1* which may have an effect on DNA dynamics, resulting in suppressed proliferation and also impacts upon the response to DNA damage (Maute et al., 2013). Mitochondrial mutation 3243A>G significantly influences tRF expression and may result in mitochondrial encephalomyopathy, lactic acidosis, and stroke-like episodes (MELAS). For example, accumulation of mt i-tRF GluUUC is dependent on modification of mt tRNAS, with mt i-tRF GluUUC down-regulating expression (like an miRNA regulator) of mitochondrial pyruvate carrier 1 (MPC1) by directly targeting the 3′ UTR of MPC1 mRNA (Meseguer et al., 2019). It has therefore been established that the miRNAs derived from tRNA can be functionally active (Maute et al., 2013). Specific tools have been developed to help identify tRFs in short RNA-seq datasets (Loher et al., 2017).

A tRF has been shown to be generated upon tRNA overexpression, and to repress target genes with a complementary sequence to the tRF in the 3′ UTR (Kuscu et al., 2018). The process is Argonaute dependent but is independent of Dicer (Kuscu et al., 2018). tRF targets mRNA pairs in the RNA induced silencing complex which associate with GW182 proteins which are involved in translational repression and in promoting the degradation of target mRNA (Kuscu et al., 2018).

## Examples of Diseases Related to Mitochondria and mtDNA

### Cardiovascular Disease (CVD)

Studies have identified a correlation between certain mtDNA polymorphisms and increased risk of developing CVD, and more recently animal models have allowed for the identification of causal relationships between mtDNA polymorphisms and cardiovascular function and pathology (Bray and Ballinger, 2017).

Mitochondrial cardiomyopathy is a severe cardiac muscle disorder occurring in the absence of coronary artery disease or hypertension that are more usually associated with myocardial dysfunction. Mitochondrial genomic defects affect the respiratory chain and are associated with abnormal heart muscle structure and/or function (Meyers et al., 2013). Mitochondrial disease can manifest as cardiac conditions such as hypertrophic and dilated cardiomyopathy, arrhythmias, and heart failure (Meyers et al., 2013).

Methylation in the mitochondrial genome may also play a role in disease, as it has been found that individuals with CVD display higher methylation levels in a number of mitochondrial genes in platelets than is typically found in healthy individuals (Baccarelli and Byun, 2015). Platelets are known to play a significant role in CVD, and also have a high ATP turnover rate (Baccarelli and Byun, 2015); therefore mitochondria are vital for platelet function, and dysfunctional mitochondria are liable to negatively affect platelet function which may result in increased CVD risk (Baccarelli and Byun, 2015).

Cardiovascular disease, resulting in reduced cardiovascular function, is associated with acute kidney injury (AKI), which results in renal dysfunction, for which there are limited treatment options and may result in a patient requiring renal replacement therapy (RRT) (Bhargava and Schnellmann, 2017). Patients requiring RRT display a mortality rate of over 60%, therefore mitochondrial genomic features which result in CVD may also result in kidney disease, which can have severe consequences for the patient (Bhargava and Schnellmann, 2017).

### Kidney Disease

Differential methylation of genes that affect mitochondrial function is associated with kidney disease in individuals diagnosed with type 1 diabetes (compared to controls) (Swan et al., 2015).

Of the 51 genes displaying significant association with diabetic kidney disease, 46 of the top ranked variants are also differentially methylated in individuals diagnosed with end-stage renal disease, with the largest changes in methylation occurring in the *TAMM41, PMPCB*, *TSFM*, and *AUH* genes (Swan et al., 2015). Epigenetic modifications of the mitochondrial genome may be contribute to kidney disease (Swan et al., 2015).

A mutation in the mitochondrial genome (m.547A>T) has been identified in patients diagnosed with maternally inherited tubulointerstitial kidney disease (Connor et al., 2017). This mutation does not result in patients displaying easily recognisable features of mitochondrial disease, but individuals with this mutation display fibroblasts with decreased levels of mitochondrial tRNA^Phe^, tRNA^Leu1^ and reduced mitochondrial protein translation and respiration (Connor et al., 2017). Other samples with the same phenotype showed a mutation in mitochondrial tRNA^Phe^ (m.616T>C) (Connor et al., 2017). These mutations are likely to cause disease as a result of a reduction in the function of mitochondrial tRNA^Phe^ (Connor et al., 2017).

### Alzheimer’s Disease

Mitochondria play a significant role in the pathology of AD, as dysfunctional mitochondria result in the generation of abnormal levels of ROS, which causes damage to neurons and cell death (Kwon et al., 2016). Additionally, dysfunctional mitochondria may result in inhibited metabolism in the brain, as abnormal metabolism may result in the accumulation of amyloid-β (Aβ) (Selkoe, 2008) and hyperphosphorylated Tau protein which result increased mitochondrial dysfunction (Khan and Bloom, 2016). This is evidenced by the observation that AD is associated with reduced cerebral glucose metabolism many years before cognitive deficits appear (Sekar et al., 2015). Studies have suggested that treatments which localise in the mitochondria could potentially supress the neuronal death which is associated with AD (Kwon et al., 2016).

Whilst established causes of AD include mutations in the nuclear genome such as those in the *AβPP* (Kalimo et al., 2014), *PSEN1* (Kelleher and Shen, 2017), and *PSEN2* genes (Jayadev et al., 2010), the mitochondrial genome and nuclear genes related to mitochondrial function have also been linked to AD. For example, differential expression of nuclear encoded – mitochondrial related genes has been identified in patients diagnosed with AD (compared to controls) including *TRMT61B, FASTKD2*, and *NDUFA4L* (Sekar et al., 2015). AD patients display a significantly higher rate of mutations in the mitochondrial genome compared to controls, including the T414G, T477C, T146C, T195C (Coskun et al., 2004), and an increased prevalence of the common mtDNA deletion mtDNA4977 (Corral-Debrinski et al., 1994). Numerous point mutations within the mitochondrial genes *CO1, CO2*, and *CO3* have been identified in AD patients, which combined with the observation that AD brains show significantly decreased CO activity compared to controls suggests that mutations within the mitochondrial genome may play a significant role in the pathogenesis of AD (Hamblet et al., 2006). AD patients also display differences in mtDNA methylation compared to controls, however, the impact of the epigenetic difference has yet to be established (Blanch et al., 2016).

### Parkinson’s Disease

Mutations in the *PINK1* gene result in early-onset PD (Tufi et al., 2014). Mitochondrial dysfunction occurs when *PINK1* is mutated; this has been shown to be a central mechanism of PD pathogenesis (Tufi et al., 2014). *PINK1* mutants undergo changes in transcription, specifically an upregulation of genes involved in nucleotide metabolism which are vital for neuronal mtDNA synthesis (Tufi et al., 2014). This was originally found in studies on *Drosophila melanogaster* but was also found in the brains of human PD patients which had *PINK1* mutations (Tufi et al., 2014). It was found that the mitochondrial dysfunction caused by *PINK1* mutation could be offset using pharmacological approaches, and also in *Drosophila melanogaster* genetic modification of the nucleotide salvage pathway offset mitochondrial dysfunction (Tufi et al., 2014). Enhancing nucleotide synthesis pathways could reverse mitochondrial dysfunction and rescue neurodegeneration of PD patients, and also potentially for patients with other diseases related to mitochondrial dysfunction (Tufi et al., 2014).

### MT tRNAs in Parkinson’s

Mutations in mitochondrial tRNAs have been linked to PD such as point mutations in the genes tRNA(Thr) and tRNA(Pro) (Grasbon-Frodl et al., 1999), and mutations in *WARS2*, which encodes mitochondrial tryptophanyl−tRNA synthetase, has been linked to infantile onset Parkinsonism (Burke et al., 2018). Additionally, PD patients display differential levels of tRFs in the frontal cortex, cerebrospinal fluid, and serum, compared to controls (Magee et al., 2019).

### Ageing

Mitochondria are involved in many processes other than ATP production, such as gene expression and epigenetics (Diot et al., 2016). Mitochondrial dysfunction tends to occur with ageing (Fang et al., 2016), which may negatively affect the various processes with which mitochondria are involved (Diot et al., 2016). Signalling from the nucleus to the mitochondria (NM signalling) is important in regulating mitochondrial function and ageing (Fang et al., 2016). Nuclear DNA damage is an initiator of NM signalling; this damage accumulates over time and may be a contributing factor in the onset of diseases associated with ageing (Fang et al., 2016). Pharmacological methods may provide a means of modulating NM signalling, thereby regulating mitochondrial function, in order to prevent and treated age associated diseases (Fang et al., 2016). Mitochondria produce ROS as a by-product of aerobic respiration, these are highly reactive and as a result cause oxidative damage to the cellular macromolecules, and as a result may cause or contribute to the ageing process (Bratic and Larsson, 2013). It has been observed that mitochondria produce a larger quantity of ROS in older individuals, and also that mutations accumulate in mtDNA as individuals’ age (Bratic and Larsson, 2013). Somatic mtDNA mutations can cause a reduction in respiratory chain function, which results in increased ROS production and further oxidative damage (Bratic and Larsson, 2013). In addition to somatic mutations, mtDNA mutations can be inherited maternally, and may play a role in the ageing process (Ross et al., 2013). Therefore, mitochondria are thought to play a significant role in the oxidative stress that drives ageing.

### Cancer

The link between mitochondrial genomic mutations and cancer was illustrated by a number of studies, such as one which observed that a 822 bp mtDNA deletion was more commonly found in cases, and is associated with a significantly increased risk of developing lung cancer (Zheng et al., 2012). Further evidence comes from a study demonstrating that mutations within the NADH dehydrogenase subunit 6 (*ND6*) gene; a missense G13997A mutation in the A11 mouse cell line, and also a frameshift 13885insC mutation in B82M mouse cells, causes defects in the electron transport chain complex I that result in increased ROS production and high metastatic potential (Ishikawa et al., 2008). It has also been observed that human MDA-MB-231 cells possess mtDNA mutations that result in defects in the electron transport chain complex I, increased ROS production, and high metastatic potential, although in this instance the mutation was not identified (Ishikawa et al., 2008). The mechanism by which these mutations confer high metastatic potential has not been proven, although it was observed that in the A11 cells the expression of the genes MCL-1, HIF-1a, and VEGF were up-regulated (Ishikawa et al., 2008).

In a study involving human breast carcinoma MDA-MB-231 cells, which display high metastatic potential, inhibited mitochondrial respiration and increased ROS production; replacing the MDA-MB-231 cells mtDNA with mtDNA found in normal human cells resulted in improved mitochondrial respiration and reduced metastatic potential, but did not affect ROS production (Imanishi et al., 2011). Mutations relating to mitochondrial tRNAs appear to have significance for a variety of diseases, although the mechanisms are poorly understood (Yarham et al., 2010). It has been suggested that mutations may affect Mt-tRNA folding and therefore inhibits tRNA maturation and may also inhibit maturation of nearby neighbouring gene transcripts (Yarham et al., 2010).

Mitochondrial tRNA and nuclear tRNA expression differs significantly in cancer cells compared to controls (Pavon-Eternod et al., 2009). In patient derived breast tumour samples, it was found that mitochondrial tRNAs expression is up-regulated by up to 13-fold compared to controls, whereas nuclear tRNAs expression increased by up to 20-fold (Pavon-Eternod et al., 2009). The expression profile of mitochondrial tRNAs has been found to vary between cell lines, whereas in contrast the expression profile of nuclear tRNAs is largely similar across cell lines (Pavon-Eternod et al., 2009).

Mutations in the mitochondrial tRNAs *tRNA^Ala^ T5655C, tRNA^Arg^ T10454C, tRNA^Leu(CUN)^ A12330G, tRNA^Ser(UCN)^ T7505C*, and *tRNA^Rhr^ G15927A* have been observed in lung cancer patients but appeared to be absent in controls (He et al., 2016). Such mutations may result in inhibited tRNA metabolism given that the mutations are found in conserved nucleotides that are necessary for rRNA steady state level. Of the aforementioned mutations T5655C, A12330G, T7505C, and G15927A can be considered as certainly pathogenic whilst T10454C may be a neutral polymorphism (He et al., 2016).

The polymorphism *tRNA*^Leu(CUN)^ A12308G has been observed in both colorectal tumour tissues and in controls, but appeared significantly more frequently in cancerous tissue (Mohammed et al., 2015). It has been suggested that this polymorphism could be considered pathogenic if found in conjunction with certain other mitochondrial conditions (Mohammed et al., 2015). However, given the mutation was found in controls, and the same size in question was small, this cannot be considered definitive.

Mt tRNA have sequence variants and/or generate tRFs that are linked to various cancers (Huang et al., 2018; Telonis et al., 2019). Telonis et al. (2019) reported that a disproportionate fraction (in terms of diversity) of distinct tRFs, from datasets in The Cancer Genome Atlas, derive from mitochondrial tRNAs compared to nuclear tRNAs. tRF–mRNA correlations are enriched in specific cancers and vary depending on sex with regards to bladder, lung, and renal cancer (Telonis et al., 2019). tRFs may have a role in drug response, for example tRFs are up-regulated in individuals with Trastuzumab-Resistant breast cancer compared to individuals who are sensitive to Trastuzumab (Sun et al., 2018). Distinctive profiles of tRNA−derived fragments have been detected in uveal melanoma. Individuals with a higher proportion of 18-nt-long tRFs and a lower proportion of 20-nt-long tRFs appear more likely to develop metastases than other individuals, and the abundance of specific tRFs appear to be associated with specific clinical stages, metastasis, and sex (Londin et al., 2019). This may vary across different cancer types, given that 18–20 nt 5′-tRFs are found more commonly in breast cancer samples than controls, whereas prostate cancer patients are less likely to have such short length tRFs than controls (Magee et al., 2018). 3′-tRFs derived from mitochondrial tRNAs are more likely to be shorter in controls, whereas prostate tumour samples tend to have a higher frequency of longer tRFs (Magee et al., 2018). The profiles of tRF, and also isomiR, appear to differ between prognostic groups based on Gleason score (Magee et al., 2018). Of particular interest to this review is the observation that of the total number of tRNA fragments found in prostate tumour samples, approximately one third are derived from mitochondrial tRNAs. Mitochondrial-derived tRFs, and isomiRs, may be involved in triple negative breast cancer (TNBC), and may explain some of the racial disparities observed with TNBC (Telonis and Rigoutsos, 2018). Whilst in normal breast tissue mRNA and non-coding RNA levels are cohesive in TNBC the mRNAs frequently display differential expression in conjunction with isomiR or tRF dysregulation (Telonis and Rigoutsos, 2018). This has consequences for a variety of pathways including those relating to energy metabolism, cell signalling, and the immune response (Telonis and Rigoutsos, 2018). In cancerous tissues these associations may be absent in many cases. It may be the case that isomiRs and tRFs have significant roles in regulatory activities which are lost in TNBC (Telonis and Rigoutsos, 2018).

tRF length and abundance may play a role in clinically relevant attributes (Londin et al., 2019). IsomiRs also display differences with regards to patient survival outcomes and disease progression, and also with metastases development (Londin et al., 2019).

## Difficulties Related to the Study of Mitochondrial Genomics

There are particular considerations relating to the mitochondrial genome, such as non-unique regions of DNA sequence, heteroplasmy, and mitochondrial haplogroups, which are not adequately accounted for by many resources, which are typically designed for use in conjunction with the nuclear genome.

Many mtDNA mutations are heteroplasmic, i.e., these mutations are not found in every mitochondrion in every cell of the affected individual. Many of the mitochondria in these cells may have normal genomes (Ye et al., 2014); mtDNA variants may only be clinically relevant once a certain threshold of heteroplasmy has been surpassed (Rossignol et al., 2003; Bris et al., 2018; Ng et al., 2018). Resources for mitochondrial genomics often lack information regarding the heteroplasmic threshold required for disease phenotypes to become evident, with MITOMAP being a notable exception (Brandon et al., 2005; Bris et al., 2018).

Combinations of polymorphisms may also have clinical relevance (Caporali et al., 2018). No computational tools exist with which to determine if an individual possesses a combination of rare mtDNA variants associated with disease, and also do not provide a means of identifying the co-occurrence of relevant nuclear genomic variants in conjunction with mtDNA variants (Dimitriadis et al., 2014; Jiang et al., 2016; Bris et al., 2018).

Mitochondrial haplogroups should be taken into consideration given the potential for an individuals’ haplogroup to have clinical relevance (Caporali et al., 2018; Klucnika and Ma, 2020). Numerous tools exist with which researchers may determine an individuals’ mitochondrial haplogroup (discussed in the “Results” section) including HaploGrep2 (Weissensteiner et al., 2016b), but databases rarely provide information for the prevalence of variants with haplogroups (Bris et al., 2018).

## MT tRNA Lookalikes

A large number of “tRNA-lookalikes” are found in the nuclear genome, i.e., sequences which are similar to human mitochondrial tRNAs, although it is notable that most appear more similar to mitochondrial tRNAs than nuclear tRNAs, with certain mitochondrial anticodons being over-represented (Telonis et al., 2014). These tRNA-lookalikes are found in close proximity to nuclear tRNAs and in some cases are transcribed as a part of other RNAs (Telonis et al., 2014). Numerous tRNA lookalikes appear to be transcribed, which appears to occur in a cell dependent manner, and the tRNA lookalikes seem to co-localise with known tRNAs. This may suggest that tRNA lookalikes give rise to functional tRNAs which play a role in biological processes, although any potential functional roles have yet to be proven (Telonis et al., 2015a). It may be of interest that tRNA-lookalikes are found in other species, particularly in other primate species (Telonis et al., 2015a), and also that in genetic variants associated with disease tRNAs tend to be enriched, whereas tRNA-lookalikes tend to be depleted (Telonis et al., 2014).

## Technical Issues

Technical limitations may restrict the use of the mitochondrial genome. High-density SNP arrays may lack mitochondrial SNPs entirely, or contain limited numbers of mitochondrial SNPs. There are difficulties associated with mapping the mitochondrial genome due to the fact mtDNA exists in numerous copies per cell that results in difficulty in implementing linkage-mapping methods based on inducing random mutations. Many bioinformatics tools developed for use with nuclear DNA deliver inconsistent and often poor results when used to analyse mtDNA, demonstrated by the finding that many of these tools predict considerable numbers of known pathogenic mutations to be benign (Bris et al., 2018). The ability to modify the mitochondrial genome is also limited (Klucnika and Ma, 2020).

The field of mitochondrial bioinformatics appears to lack tools in a number of areas such as gene expression, tRNAs, tRFs, and epigenetics. Development of appropriate tools in these areas may provide researchers with a means of exploring neglected areas relating to the mitochondrial genome, which may provide a means of improving understanding of the genetic basis of disease. Improved understanding may result in clinical benefits such as more effective genetic counselling and optimal mitochondrial replacement therapies.

### tRNA and tRFs

Researchers investigating tRNA and tRFs may consider using tools such as MITOMAP (Lott et al., 2013), MINTbase (Pliatsika et al., 2018), and MINTmap (Loher et al., 2017). Approximately half of mtDNA alterations relate to tRNAs (Schaefer et al., 2008; Gorman et al., 2015). Given that tRFs have roles in a variety of biological processes (Telonis et al., 2019) and may be relevant with regards to cancer (Maute et al., 2013; Magee et al., 2018), tools which further enable research relating to tRNAs and tRFs may have clinical benefits.

### Gene Expression Resources

Other than the tools relating to tRNA, the discussed resources do not provide functions relating to gene expression (Van Dang et al., 2017). Future resources may wish to consider topics such as the role of mitochondrial haplogroups and gene expression (Krijger and de Laat, 2016; Van Dang et al., 2017; Schneider et al., 2018).

### Epigenetics

To the authors’ knowledge, no software is available for the investigation of mitochondrial epigenetics. While it must be noted that published literature disagrees regarding the existence of mtDNA methylation, it could be suggested that insufficient research has been carried out on this topic from which a definitive conclusion may be drawn (Pirola et al., 2013; Baccarelli and Byun, 2015; Mechta et al., 2017). The lack of suitable software tools may play a role in such uncertainty.

### Future Development of Tools

The difficulties facing the field of mitochondrial genomics relate to certain intrinsic difficulties associated with the mitochondrial genome which will require further progress in the wet laboratory techniques, such as the development of effective genetic modification methods (Klucnika and Ma, 2020). Progress in this area will facilitate further research into mitochondrial genomics, which will also require adequate bioinformatics tools. Other technical improvements such as including more mitochondrial SNPs on SNP arrays would facilitate the increased study of the mitochondrial genome in GWAS.

Overcoming the current obstacles in the field of mitochondrial genomics would facilitate further research into the impact of the mitochondrial genome on human diseases, potentially resulting in improved genetic counselling and medical treatments.

## Results

### Online Mitochondrial Resources

**HmtDB** accessible at **https://www.hmtdb.uniba.it/**

**Programming ability required:** NA.

**Data input:** NA.

**Citation counts:** Original 2005 article – 48 citations, updated 2011 article – 91 citations, updated 2016 article – 16 citations.

HmtDB is an online database of annotated human mitochondrial genome sequences, which includes population data, and nucleotide and amino acid variability data (Attimonelli et al., 2005). The database was designed to support research regarding population genetics and mitochondrial disease. The mtDNA sequences were assembled from a number of sources such as the International Nucleotide Sequence Database Collaboration (INSDC), and from application of the MToolBox pipeline to NGS data such as that obtained from the 1000 Genomes project. The database contains 28196 healthy genomes and 3539 pathologic genomes, each of which are divided into regional groups such as African, Asian, European, etc. (Rubino et al., 2012).

HmtDB also allows for query sequences submitted by the user to be compared with the reference sequences contained within the database, which can be useful for predicting the pattern of SNPs in the query sequence (Rubino et al., 2012).

The genomes contained in HmtDB are analysed using the Variability Generation Work Flow (VGWF) system, which enriches sequence information with variability data estimated using algorithms (Attimonelli et al., 2005). Additionally, the classification work flow system is used to classify newly sequenced genomes (Attimonelli et al., 2005).

HmtDB has been used in various studies, such those investigating links between genetic variation and breast cancer, which used HmtDB as a reference with which to compare the quality of sequences and coherence of variants obtained during the study (Tommasi et al., 2014). HmtDB has also been used as a source of mitochondrial genomes for use as part of other studies, for example mitochondrial genomes downloaded from HmtDB were used as part of study which aimed to determine if mtDNA is under selection pressures (Piredda et al., n.d.).

**MToolbox** accessible at **https://github.com/mitoNGS/MToolBox** and also **https://mseqdr.org/**

**Programming ability required:** None for GUI version provided at MSeqDR, command line ability required in order to use github version.

**Data input:** One of either FASTA, FASTQ, FASTQ.GZ, BAM, or SAM format.

**Citation count:** 90.

MToolBox is an automated pipeline for analysing mtDNA sequences obtained through NGS which provides a variety of information including the sequences’ genotype, haplogroup assignment, and variant prioritisation. A use for this tool includes identifying mitochondrial genomic mutations, which may be associated with disease (Calabrese et al., 2014). MToolBox contains a number of tools, including classifier tools that can be used to predict the haplogroup of mitochondrial genomes using haplogroup definitions based on the Phylotree system. These tools can be applied to genomes contained within the database and can also be applied to mitochondrial genomes submitted by the user. The tool may be used either in its’ online form or else downloaded by the user (Calabrese et al., 2014).

MToolBox has been employed in numerous studies, with uses including mitochondrial single nucleotide variant calling in a study relating to prostate cancer (Hopkins et al., 2017), and to extract mitochondrial reads, perform variant calling, and identify haplogroups in a study relating to Autism spectrum disorder (Patowary et al., 2017).

**mitotRNAdb** accessible at **http://mttrna.bioinf.uni-leipzig.de/mtDataOutput/**

**Programming ability required:** NA.

**Data input:** NA.

**Citation count:** 618.

mitotRNAdb is a database containing over 30,000 mitochondrial tRNA genes, drawn from over 1,500 metazoan mitochondrial genomic sequences drawn from RefSeq, which are classified based on amino acid specificity (Juhling et al., 2009). The resource is utilised by either browsing a taxonomic tree or alternately by utilising the search function, which may be refined based on DNA and RNA sequences, the amino acid family, gene ID, references, and structural characteristics. Users may also download the sequences provided by mitotRNAdb (Juhling et al., 2009).

mitotRNAdb has been utilised in a considerable number of studies, providing researchers with information required for a wide array of studies. For example, mitotRNAdb was utilised in the design of DNA probes in a study relating to bacterial tRNA modification (Reichle et al., 2019), and also provided reference information in a study which investigated the evolution of tRNA (Zamudio et al., 2019).

**MitoCarta 2.0** accessible at **https://www.broadinstitute.org/scientific-community/science/programs/metabolic-disease-program/publications/mitocarta/mitocarta-in-0**

**Programming ability required:** NA.

**Data input:** NA.

**Citation count:** 592.

MitoCarta 2.0 (also termed MitoCarta: An Inventory of Mammalian Mitochondrial Genes) consists of a database of 1158 human genes and 1158 mouse genes, all of which encode proteins associated with mitochondrial localisation. These genes were identified through laboratory procedures and the results of this were integrated with six different datasets of mitochondrial localisation using a Bayesian approach (Calvo et al., 2016).

MitoCarta 2.0 also includes a dataset containing images of 131 proteins for which mitochondrial localisation was assessed using mass spectrometry, green fluorescent protein tagging, and microscopy (Krasich and Copeland, 2017), and datasets indicating scores of mitochondrial localisation for over 20,000 human genes and 20,000 mouse genes, and datasets of human and mouse protein sequences (Calvo et al., 2016). MitoCarta 2.0 therefore serves as a reference database but does not include analytical tools accessible to users (Calvo et al., 2016).

Mitocarta 2.0 has been used as a reference database of nuclear encoded genes which are involved with mitochondria (in a study investigating mitochondrial genome in relation to cancer) (Chattopadhyay et al., 2017), and has also been used as a reference with which the results of studies can be compared, such as protein subcellular localisation predictions (Huttlin et al., 2017).

**MitoBreak** accessible at **http://mitobreak.portugene.com/cgi-bin/Mitobreak_home.cgi**

**Programming ability required:** NA.

**Data input:** Manual input of breakpoint locations.

**Citation count:** 28.

Mitobreak contains curated datasets of mtDNA rearrangements and includes analytical tools which are accessible by users (Damas et al., 2014).

The datasets include data relating to various types of mtDNA rearrangements including Circular deleted mtDNAs, circular partially duplicated mtDNAs, and linear mtDNAs, from various species including human, house mouse, Norway rat, and fruit fly. These datasets can be downloaded by users, and users can submit data to Mitobreak making it accessible to others (Damas et al., 2014).

Mitobreak functions include statistical tools used to investigate the datasets of mtDNA breakpoints, and a comparison system allowing breakpoints identified via the users own research to be compared with those found in Mitobreak datasets (Damas et al., 2014).

Mitobreak has been used as a source of deletion data relating to the mitochondrial genome, in a study investigating links between mtDNA deletions and disease (Bharti et al., 2014), and also in a study relating to the measurement of mtDNA deletion levels and copy number differences (Grady et al., 2014). Mitobreak has also provided data for a study which aimed to identify experimental assay parameters which would allow for the analysis of low levels of deletion in the mitochondrial genome (Belmonte et al., 2016).

**MSeqDR (Mitochondrial Disease Sequence Data Resource Consortium)** accessible at **https://mseqdr.org/**

**Programming ability required:** Not required for web browser use, although users may use programmatic methods to retrieve data.

**Data input:** Includes tools compatible with VCF from WGS or WES, clinical data, and also provides tools compatible with FASTA, FASTQ, FASTQ.GZ, BAM, or SAM format data.

**Citation counts:** 2015 article – 56, 2016 article – 18, 2018 article – 9.

MSeqDR is a resource that collects and shares data relating to rare diseases and genetic mutations known/believed to cause these diseases. This includes data relating to 183 diseases and 1568 genes. MSeqDR also includes various tools which allow the user to perform a number of tasks, including (MSeqDR Consortium, 2019):

Disease portal – A mitochondrial disease can be selected and known symptoms, associated genes and variants (MSeqDR Consortium, 2019),

HPO browser – The human phenotype ontology tree can be browsed (MSeqDR Consortium, 2019),

Human BP Codon Resource Variant Annotation Pipeline (HBCR) – Allows functional annotation of variants (MSeqDR Consortium, 2019),

myTool – Multiple mtDNA variant formats can be converted into one standard format (MSeqDR Consortium, 2019),

MSeqDR has been used as a reference database of missense changes in a study relating mitochondrial cytochrome b variants (Song et al., 2016).

**MITOMAP** accessible at **https://www.mitomap.org/MITOMAP**

**Programming ability required:** Required for certain functions. Sample scripts in Perl and Python are provided.

**Data input:** FASTA or text file.

**Citation counts:** 1996 article – 147, 1997 (update) article – 32, 1998 article – 157, 2005 article – 420, 2006 article – 577.

MITOMAP is a database of known polymorphisms and mutations in human mtDNA, which allows users to add their own papers and data. MITOMAP contains a variety of data types, including pathological mutations in transfer RNA (tRNA), population migrations, diabetes metabolism and mitochondria, annotated human mtDNA sequence, amino acid translation tables, and mitochondrial genome sequences. This tool potentially serves a broad audience due to the variety of data it contains (Lott et al., 2013). MITOMAP has been used as a reference database with which samples taken by researchers were compared, in a study investigating the mitochondrial genome in relation to type 2 diabetes mellitus, atherosclerosis and essential hypertension (Ding et al., 2016).

**MitoMiner 4.0** accessible at **http://mitominer.mrc-mbu.cam.ac.uk/release-4.0/begin.do**

**Programming ability required:** Not required for all functions, however, users may programmatically acquire data from MitoMiner 4.0 using Perl, Python, Ruby, or Java scripts.

**Data input:** Text.

**Citation counts:** 2015 article – 93, 2018 article – 10.

A resource that contains and analyses mitochondrial proteomics data (Smith and Robinson, 2019), including evidence indicating where specified proteins are found in the mitochondria, phenotypes, interactions, metabolic pathways, and disease information (Smith and Robinson, 2019). This resource integrates proteomics data gathered in various studies *via* green fluorescent protein tagging and mass spectrometry, with data from UniProt, Gene ontology, Online Mendelian Inheritance in Man, HomoloGene, Kyoto Encyclopaedia of Genes and Genomes and PubMed (Smith and Robinson, 2019).

Two different sets of reference genes are used – MitoCarta 2.0 – contains 1158 human and mouse genes which encode proteins which support mitochondrial localisation (Smith and Robinson, 2019).

Uses of MitoMiner 4.0 include the investigation of whether a gene encodes a mitochondrial protein, the function of a gene, the expression of a gene specifically in certain tissues, the variability of the mitochondrial proteome between different tissues, the knockout phenotype of genes (in mice, zebrafish, and yeast), the investigation of genes which interact with a given gene, the study of protein associations with disease, and the effects of changes in the proteome on mitochondrial disease (Smith and Robinson, 2019).

MitoMiner 4.0 has been used as an analytical tool to determine the localisation of particular proteins in the mitochondria of human stem cells (Shekari et al., 2017),and also to assess the isolation of mitochondrial proteins in a study relating to mitochondrial proteomic changes associated with PD (Villeneuve et al., 2016).

**The mitochondrial proteome (IMPI)** accessible at **http://www.mrc-mbu.cam.ac.uk/impi**

**Programming ability required:** NA.

**Data input:** NA.

**Citation counts:** NA (note those who cite MitoMiner 4.0 may be using data provided by IMPI).

IMPI can be accessed *via* MitoMiner 4.0. MitoMiner 4.0 combines data provided by IMPI and MitoCarta 2.0, therefore overlap exists between the data provided by MitoMiner 4.0 and that provided by IMPI. Consists of a database of genes that encode proteins with evidence for cellular localisation within the mammalian mitochondrion, based on a variety of evidence such as metabolomics models, antibody data, and GFP and mass spectrometry localisation studies. In total, the database contains data regarding 1408 genes encoding proteins that are localised in the mitochondria. This resource has been used to identify disease-causing genes that were not previously known to be mitochondrial (IMPI, 2015).

IMPI has been used to compile a mitochondrial proteome, which was used to study the role of common genetic variations in disease (Johnson et al., 2017).

**MtDNA-Server** accessible at **https://MtDNA-server.uibk.ac.at/index.html**

**Programming ability required:** NA.

**Data input:** FASTQ (single or paired end), or BAM/SAM.

**Citation count:** 56.

Carries out automated analysis of human mtDNA, with a focus on the identification of heteroplasmy and contamination within mtDNA samples and can do so in a parallelised manner. Includes filters relating to sequence error, quality, and statistical methods used in relation to determining heteroplasmy (Weissensteiner et al., 2016a).

MtDNA-Server provides users with the ability to assign appropriate haplogroups, detect heteroplasmy, detect contamination/artefacts, annotate variants, operate quality control procedures, and to assemble the mitochondrial genome (Weissensteiner et al., 2016a).

MtDNA-Server has been used to analyse genetic sequence data in a study regarding mitochondria in relation to prostate cancer (Schopf et al., 2016), and was also used to detect mutations in sequenced data in a study which investigated genetic variation in relation to CVD risk reduction (Coassin et al., 2017).

**Phy-Mer** accessible at **https://github.com/danielnavarrogomez/phy-mer**

**Programming ability required:** Command line use required.

**Data input:** CSV, FASTA, FASTQ, or BAM formats.

**Citation count:** 34.

A tool used to classify mitochondrial haplogroups, which is automated and does not require alignment and which does not require a reference sequence (Navarro-Gomez et al., 2015). This contrasts with many other haplogroup classification tools which identify variants based on an alignment from a reference sequence, and which require the variant to have been named previously, and then compare the identified variants with polymorphisms which determine haplogroups (Navarro-Gomez et al., 2015). NGS data can be used as direct input into Phy-Mer, and Phy-Mer has also been shown to be less error prone than HaploGrep (a commonly used haplogroup classifier) (Navarro-Gomez et al., 2015).

Phy-Mer was used to determine the haplogroup of a number of individuals in a study which aimed to identify human remains based on the genetic data of known living relatives (Fernandes et al., 2017).

**HaploGrep2** accessible at **https://haplogrep.i-med.ac.at/download-2/** and **https://github.com/seppinho/haplogrep-cmd**

**Programming ability required:** Ability to use command line.

**Data input:** VCF.

**Citation count:** 198.

A tool used for the haplogroup classification of mtDNA, in which haplogroup classification is based on Phylotree. HaploGrep2 accepts a variety of input file formats, and the process is automated, making the tool convenient to use. The output displays the top results, the phylogenetic position of the respective haplogroup, and explains how and why the haplogroup was selected. Visualisation of the results is also provided, as are suggestions of which polymorphisms should be additionally analysed in order to improve the accuracy of the result (Weissensteiner et al., 2016b).

HaploGrep2 provided automated investigation of sample contamination, as part of a method for processing mitochondrial genomes sequenced using NGS (Weissensteiner et al., 2016b).

**HAPLOFIND** accessible at **https://haplofind.unibo.it/**

**Programming ability required:** NA.

**Data input:** FASTA.

**Citation counts:** 98.

An mtDNA haplogroup assignment tool that uses information phylogeny information from Phylotree and information from GenBank regarding the conserved mutations of many mtDNA sequences. This tool can also detect mutations and identify whether these are associated with disease (Vianello et al., 2013).

HAPLOFIND has been used to assign a haplogroup to patients involved in a study regarding a mitochondrial genomics variant associated with reduced embryonic aneuploidy risk (Olcha et al., 2015).

**H-mito** accessible at **https://sourceforge.net/projects/h-mito/**

**Programming ability required:** Ability to use command line and Python required.

**Data input:** FASTA.

**Citation count:** 10.

An mtDNA haplogroup prediction software tool, based on Phylotree. H-mito extracts mutation lists and joins and aligns sequences using clustalw (a programme used to align multiple DNA or protein sequences) (Valentini et al., 2010).

**MitoSeek** accessible at **https://github.com/riverlee/MitoSeek**

**Programming ability required:** Ability to use command line.

**Data input:** BAM (paired end).

**Citation count:** 76.

This tool can be used to extract data from the whole mitochondrial genome, or alternatively just the exome. MitoSeek evaluates the mitochondrial genome alignment quality, estimates relative mitochondrial copy numbers, detects heteroplasmy, somatic mutations, and structural variants of the mitochondrial genome (Guo et al., 2013).

MitoSeek has been used to screen for mitochondrial genomic variants in a study investigating the mitochondrial genome in relation to neuroblastoma (Riehl et al., 2016).

**MITOMASTER** accessible at **https://www.mitomap.org/foswiki/bin/view/MITOMASTER/WebHome**

**Programming ability required:** NA.

**Data input:** Either text or FASTA.

**Citation count:** 65.

MITOMASTER identifies nucleotide variants and haplogroups, assesses pseudogene contamination, and also assesses the potential biological significance of the identified variants. This tool may be of use in fields such as biomedical science, population biology, and forensic science (Brandon et al., 2009).

MITOMASTER provided researchers with a means to investigate allele frequencies, by screening mitochondrial genome sequence variations found in the National Centre for Biotechnology Information (NCBI) database in a study investigating a mitochondrial tRNA variant in relation to pancreatic cancer (Li et al., 2016).

**mtDB – Human Mitochondrial Genome Database** accessible at **http://www.mtdb.igp.uu.se/**

**Programming ability required:** NA.

**Data input:** NA.

**Citation count:** 343.

The mtDB – Human Mitochondrial Genome Database is an online resource that provides links, which users can follow in order to download mtDNA sequences from a large number of different human populations, and also a limited selection of mtDNA sequences from apes. mtDB also contains a list of polymorphic sites for Cambridge Reference Sequence (CRS) nucleotide positions, including 1865 full sequences and 839 coding region sequences, and has functions for searching for specific variants at defined genomic sites, and for searching for sequences matching with mitochondrial haplogroups (Ingman and Gyllensten, 2006).

mtDB provided reference genomes in a study which reconstructed a mitochondrial genome sequence (Green et al., 2008), and also provided mitochondrial genomes for use in a study regarding selection in the transmission of mtDNA (Stewart et al., 2008).

It must be noted that mtDB has not been updated since 2007 and so may not be a reliable tool, therefore it may be advisable to employ a different, more up to date tool.

**ChloroMitoSSRDB 2.0** accessible at **http://www.mcr.org.in/chloromitossrdb/**

**Programming ability required:** NA.

**Data input:** FASTQ.

**Citation count:** 17.

This resource is a database of simple sequence repeats (SSRs), which are also known as Microsatellites, from the chloroplast and mitochondrial genomes of a wide range of species. SSRs are significant in the study of evolution and linkage analysis (the study of linkage between genes) making this a useful tool. This database allows users to access the SSRs, primer pair information, and also allows for whole genome analysis in order to identify SSRs. The users own NGS data can be uploaded for analysis, and microsatellites association with coding and non-coding regions can be visualised (Sablok et al., 2013).

**PhyloTree*_mt_*** accessible at **https://www.phylotree.org/**

**Programming ability required:** NA.

**Data input:** NA.

**Citation count:** 1456.

A website that contains a phylogenetic tree of variation in the human mitochondrial genome. Over 5400 haplogroups are included, as are the mutations that define these haplogroups (Van Oven and Kayser, 2009). PhyloTree provides an overview of the evolution of humans, based on the matrilineal line, and serves as a framework for a variety of researchers, and is used by a number of other mitochondrial resources as a basis for their own databases (Van Oven and Kayser, 2009).

PhyloTree*_mt_* was used as a reference from which to determine the evolutionary relationships among mtDNA haplogroups in a study regarding the implications of mitochondrial replacement technologies on human evolution (Rishishwar and Jordan, 2017).

**MitoAge** accessible at **http://www.mitoage.info/**

**Programming ability required:** NA.

**Data input:** NA.

**Citation count:** 10.

A database containing mtDNA data integrated with longevity records, and also includes tools for analysis of mtDNA (with a focus on this in relation to longevity). This is significant because mitochondria are believed to play a major role in ageing and age related diseases, as it has been shown that there are correlations between the mtDNA sequence and the maximum lifespan of mammals (Toren et al., 2016). Therefore, this tool may be of use in the research of longevity.

**MitoPhAST** accessible at **https://github.com/mht85/MitoPhAST**

**Programming ability required:** Ability to use command line and Unix/Linux.

**Data input:** GenBank or EMBL data.

**Citation count:** 24.

A tool used to identify annotated protein-coding gene features, and which generates an amino acid alignment from GenBank/EMBL-format mitogenome flat files, and which also generates a phylogenetic tree using optimised protein models, and reports mitochondrial genes and tDNA sequence information (Tan et al., 2015).

MitoPhAST has been used to carry out summary statistics such as gene boundaries, gene length, nucleotide composition, number of genes, etc. as part of a mitochondrial genome sequencing project involving shrimp species (Tan et al., 2017).

**MamMiBase** accessible at **http://www.mammibase.lncc.br**

**Programming ability required:** NA.

**Data input:** NA.

**Citation count:** 16.

A database (no updates from 2010 to date) used to retrieve individual gene sequence alignments for genes in complete mammalian mitochondrial genomes, for use in phylogenetics. The sequences contained in the database may be downloaded, using parameters set by the user including the sequence length, base content, etc. A phylogenetic tree can also be downloaded, which may be of use for calculating parameters (Vasconcelos et al., 2005).

MamMiBase provided coding sequences of particular mitochondrial genes for use in a study regarding the evolution of the genomic recombination rate in mammals (Dumont and Payseur, 2008).

**MINTbase 2.0** accessible at **https://cm.jefferson.edu/MINTbase/**

**Programming ability required:** NA.

**Data input:** NA.

**Citation counts:** 2016 article – 36, 2017 article – 24.

MINTbase 2.0 is a database of 26,531 tRFs from the human nuclear and mitochondrial genomes, based upon 11,719 datasets. Information is provided with regards to the genomic locations at which tRNAs could possibly be found (allowing users to avoid mistaking tRNA lookalikes and partial sequences for tRFs). Users have the ability to filter tRF data based upon criteria such as abundance thresholds, alignments between a tRNA and numerous tRFs, and the ability to generate tRF abundance plots with regard to cancers (Pliatsika et al., 2018).

MINTbase provided researchers with a means of determining whether tRFs are exclusive to tRNA space, in a study which identified tRFs as biomarkers for Trastuzumab-Resistant Breast Cancer (Sun et al., 2018). MINTbase also provided a means to tRF classification to a study which identified a mutation that alters the expression of mitochondrial tRFs (Meseguer et al., 2019).

**MINTmap** accessible at **https://github.com/TJU-CMC-Org/MINTmap/**

**Programming ability required:** Ability to use command line.

**Data input:** FASTQ.

**Citation count:** 34.

This software provides a means of identifying nuclear and mitochondrial tRFs in short RNA-seq datasets, and also calculating and reporting the abundancies of the tRFs (Loher et al., 2017). Any identified tRFs which originate from outside of tRNA space are marked as potential false positives (Loher et al., 2017).

MINTmap has been used to classify tRFs in a study relating to the role of Pseudouridylation in stem cell commitment (Guzzi et al., 2018), and to classify and count the number of reads of tRFs present in biofluids (Godoy et al., 2018).

## Discussion

### Applications of the Resources Discussed

The resources discussed serve a variety of functions (Figures 1, 2), some resources may be better suited to some tasks than others, and certain tasks may only be facilitated by one of the discussed resources. This is summarised as follows;

If the intention is to use the resource as a reference only, then those on the left column of Table 1 might be the most appropriate to consult. If the intention is to use the resource to carry out data analysis, then those on the right column of Table 1 would be most appropriate. Many of the resources have specific focuses/functions, which affects the circumstances in which they may be most useful.

**TABLE 1:** Resources that function as a database only, compared to resources which include analytical tools.

Database only	Includes analytical tools
MitoCarta 2.0	HmtDB
MITOMAP	Mitobreak
MitoMiner 4.0	MSeqDr
The mitochondrial proteome (IMPI)	MtDNA-Server
mtDB – Human Mitochondrial Genome Database	Phy-Mer
PhyloTreemt	HaploGrep2
MamMiBase	HAPLOFIND
mitotRNAdb	MtDNAoffice
MINTbase 2.0	H-mito
	MitoSeek
	MITOMASTER
	ChloroMitoSSRDB 2.0
	MitoAge
	MitoPhAST
	MINTmap

If the user is only interested in the mitochondrial genome, then the resources listed on the left of Table 2 may suffice, however, since a significant number of nuclear genes affect mitochondrial function many researchers are likely to be interested in the resources listed on the right of Table 2.

**TABLE 2:** Resources which deal with the mitochondrial genome exclusively, compared to resources that also deal with mitochondrial genes that are encoded by the nuclear genome.

Mitochondrial genome only	Includes nuclear genes related to mitochondria
HmtDB	MitoCarta 2.0
Mitobreak	MSeqDR
MITOMAP	MitoMiner 4.0
MtDNA-Server	The mitochondrial proteome (IMPI)
Phy-Mer	MINTbase 2.0
HaploGrep2	
HAPLOFIND	
MtDNAoffice	
H-mito	
MitoSeek	
MITOMASTER	
mtDB – Human Mitochondrial Genome Database	
ChloroMitoSSRDB 2.0	
PhyloTreemt	
MitoAge	
MitoPhAST	
MamMiBase	
mitotRNAdb	

More specialised functions that may not be desired by a large number of users are typically not facilitated by a large number of resources (Table 3), instead such functions tend to be provided by more specialised resources such as those listed in Table 4.

**TABLE 3:** Discussed resources in relation to functions they can perform.

Annotate mitochondrial genome	Phylogenetic studies/functions	Disease information/pathological mutations	Identification of heteroplasmy	Haplogroup classification	Identification of variants	Annotation of variants
MitoPhAST	MamMiBase	MitoMiner 4.0	MtDNA-Server	HmtDB	HmtDB	MSeqDR
	HaploGrep2	HmtDB	MitoSeek	MtDNA-Server	Mitobreak	MtDNA-Server
	PhyloTreemt	MSeqDR		Phy-Mer	MtDNA-Server	
	MitoPhAST	MITOMAP		HaploGrep2	HAPLOFIND	
		HAPLOFIND		HAPLOFIND	MitoSeek	
		MINTbase		MtDNAoffice	MITOMASTER	
				H-mito		
				MITOMASTER		
				mtDB		

*More commonly desired functions are typically facilitated by numerous resources.*

**TABLE 4:** The discussed resources in relation to more specialised functions they can perform.

Resource function	Resource	
Microsatellite identification	ChloroMitoSSRDB 2.0	
Longevity studies	MitoAge	
Mitochondrial localisation of proteins	IMPI	
Gene function	MitoMiner 4.0	
Gene expression	MitoMiner 4.0	
Gene interactions	MitoMiner 4.0	ChloroMitoSSRDB 2.0 (linkage analysis)
Metabolic pathways	MitoMiner 4.0	
Compare diseased with healthy genomes	HmtDB	
Compare population groups	HmtDB	
Assess potential biological significance of variants	MITOMASTER	
Genotype identification	HmtDB	
tRNA related	MITOMAP (mutations in tRNA)	mitotRNAdb
tRF related	MINTbase 2.0	MINTmap
		

Certain studies may require data relating to species other than humans. The majority of resources discussed relate to humans only, however, those listed in Table 5 contain data relating to other species, and so those resources may be of more use to some researchers.

**TABLE 5:** Resources that contain data relating to non-human animals and plants.

Includes non-human animals	Includes non-human animals and plants
MitoCarta 2.0	ChloroMitoSSRDB 2.0
MitoMiner 4.0	
MITOMASTER	
mtDB – Human Mitochondrial Genome Database	
mitotRNAdb	

*The other resources discussed previously but not included in this table contain data related to humans only.*

The choice of which resource to use may also be influenced by the number of reference genomes, genes, etc. contained within the resource, and also potentially characteristics of the genomes.

For example, MitoCarta 2.0 contains 1158 human genes (Calvo et al., 2016), whereas MSeqDr contains 1568 genes (MSeqDR Consortium, 2019). It may be useful to have access to as large a number of genes as possible, and therefore some researchers may prefer to use MSeqDr over MitoCarta 2.0 for this reason. However, MSeqDr focuses on genes related to disease (MSeqDR Consortium, 2019), whereas MitoCarta 2.0 is less specific and so may be more useful to users who are not researching genetic factors in disease (Calvo et al., 2016).

In some cases, the type of genes may matter more to a user than the quantity of genes, for example IMPI includes 1408 genes encoding proteins which are localised in the mitochondria (Smith and Robinson, 2016), and so may be a more useful resource to some users due to the nature of the genes, compared to MSeqDr which contains more genes but which are not specifically those which are localised in the mitochondria (MSeqDR Consortium, 2019).

Researchers investigating tRNA may use MITOMAP (Brandon et al., 2005) or mitotRNAdb (Juhling et al., 2009) as reference databases, but analytical tools in this area are lacking. A similar lack of choice faces those investigating tRFs. MINTbase provides a reference dataset regarding tRFs (Pliatsika et al., 2018) and MINTmap provides a means of identifying tRFs in the users’ data (Loher et al., 2017).

MitoCarta 2.0 contains data indicating the level of mitochondrial localisation for over 20,000 human and 20,000 mouse genes (Calvo et al., 2016), which may be more useful to some users due to the large number of genes listed, compared to IMPI which contains data relating to 1408 genes (Smith and Robinson, 2016). However, it is also possible that users may find IMPI a more useful resource than MitoCarta 2.0 because the genes listed in IMPI are all localised in the mitochondria (Smith and Robinson, 2016), whereas MitoCarta 2.0 includes a large number of genes which may not be relevant to the user as they are not localised in the mitochondria (Calvo et al., 2016).

Some users may find HmtDB a useful resource due to the large number of reference genomes (28196 healthy genomes and 3539 pathologic genomes) it contains (Rubino et al., 2012), rather than resources which are limited to a list of genes such as MitoCarta (Calvo et al., 2016), MSeqDr (MSeqDR Consortium, 2019), mtDB (Ingman and Gyllensten, 2006), etc.

A users’ choice of resource may also depend on the format of data with which the resource deals. Resources such as HmtDB (Rubino et al., 2012), and ChloroMitoSSRDB 2.0 (Sablok et al., 2013), can be used in conjunction with NGS data, whereas HaploGrep2 accepts multiple data formats (Weissensteiner et al., 2016b).

The choice of resource may also depend on whether the user simply desires a reference database, or if they also desire analytical tools. As discussed previously, resources such as MitoCarta 2.0 (Calvo et al., 2016), MITOMAP (Lott et al., 2013), and PhyloTree*_mt_* (Van Oven and Kayser, 2009), are databases which do not include analytical tools, and so may be the most appropriate resource for users who require a reference database only. Resources such as HmtDB (Rubino et al., 2012), Mitobreak (Damas et al., 2014), MSeqDr (MSeqDR Consortium, 2019), and MitoAge (Toren et al., 2016), include analytical tools and so would be more appropriate for users who wish to analyse data.

Generally, the most appropriate resource for a particular user will depend on the type of research they are carrying out and the objectives of that research. It is likely that none of the resources discussed can individually provide every user with the data and tools they require, therefore the user must decide which resources are most suitable for their work, and it is possible that a user would need to use multiple resources if no one resource provided all the data and tools they required.

## Materials and Methods

The online searches were carried out using Google, Google Scholar, and PubMed, using multiple keywords, in an attempt to identify any existing review article of mitochondrial genomic tools. No such article was identified; therefore, the decision was taken to write this review.

The information contained in this review was obtained via the search engines Google, Google Scholar, and PubMed. The relevant resources and associated papers were accessed; the resources were then discussed based on the information contained therein.

## Author Contributions

RC performed literature searches and drafted the manuscript. AJM provided guidance regarding content, formatting, and editing. APM and CC provided guidance regarding formatting and editing.

## Conflict of Interest

The authors declare that the research was conducted in the absence of any commercial or financial relationships that could be construed as a potential conflict of interest.
